# Vomiting-Induced Gastric Emphysema and Hepatoportal Venous Gas: A Case Report and Review of the Literature

**DOI:** 10.1155/2015/413230

**Published:** 2015-02-11

**Authors:** Malav P. Parikh, Muhammed Sherid, Venu Ganipisetti, Venu Gopalakrishnan, Maria Habib, Monika Tripathi

**Affiliations:** ^1^Division of Gastroenterology and Hepatology, Department of Internal Medicine, Presence Saint Francis Hospital, Evanston, IL 60202, USA; ^2^Department of Gastroenterology and Hepatology, Georgia Regents University, Augusta, GA 30912, USA; ^3^Department of Radiology, Presence Saint Francis Hospital, Evanston, IL 60202, USA

## Abstract

Gastric pneumatosis is the presence of air within the wall of the stomach. It represents a spectrum of conditions ranging from benign disease to septic shock and death. Etiopathologically, it can be classified into emphysematous gastritis or gastric emphysema (GE). Along with hepatoportal venous gas (HPVG), it was considered as an ominous radiological sign and warranted an emergent surgical exploration; however, with widespread use of computerized tomographic (CT) scan, an increasing number of benign causes of GE and HPVG have been reported in the literature, where patients can be managed by noninvasive and conservative measures. We hereby describe a case where recurrent episodes of vomiting led to development of GE and HPVG and the patient was managed successfully by conservative measures.

## 1. Introduction

Gastric pneumatosis refers to the presence of air within the wall of the stomach. It can be classified into two types: gastric emphysema (GE) and emphysematous gastritis. GE is essentially non-life threatening and can be caused by a variety of iatrogenic and noniatrogenic events [1–6]. Pulmonary, trauma, and obstruction are the three principal theories proposed to explain its pathogenesis. They have a benign clinical course and patients often have uneventful recovery with conservative treatment [1].

In rare instances, air within the stomach wall can dissect through the tissue planes and reach the portal veins, leading to hepatoportal venous gas (HPVG). Now from a radiological stand point HPVG denotes a catastrophic abdominal event like intestinal necrosis or bowel obstruction mandating urgent surgical intervention. However with growing use of CT scans in recent times, several benign cases of HPVG are described in the literature where invasive surgical approach is often not required and patients can be managed conservatively. CT scan plays an important role in such cases of acute abdomen to identify the underlying etiological process and help the clinician choose conservative over surgical treatment option [6–8]. We hereby present a case of GE and HPVG induced by vomiting where patient was successfully managed by conservative measures. Also presented is a review of the literature to shed a light on etiology, clinical presentation, and management strategies in cases of GE, emphysematous gastritis, and HPVG.

## 2. Case Presentation

A 58-year-old male presented to the emergency room with complaints of abdominal pain, nausea, and multiple episodes of vomiting for last twenty-four hours. Patient described a sharp epigastric pain with 8–10 episodes of bilious vomiting and retching. He denied eating unusual food or taking over the counter pain medications. Patient had regular bowel movements and denied any blood in the stool. His past medical history included end stage renal disease requiring hemodialysis and peripheral vascular disease.

On physical examination, pulse was 90 beats per minute, blood pressure was 150/90 mm of Hg, and temperature was 98.6° F. Mild tenderness was present in the epigastric region, but abdomen was otherwise soft and without guarding or rigidity. Fecal occult blood test was negative. The remainder of the physical exam was unremarkable.

**Figure 1:**
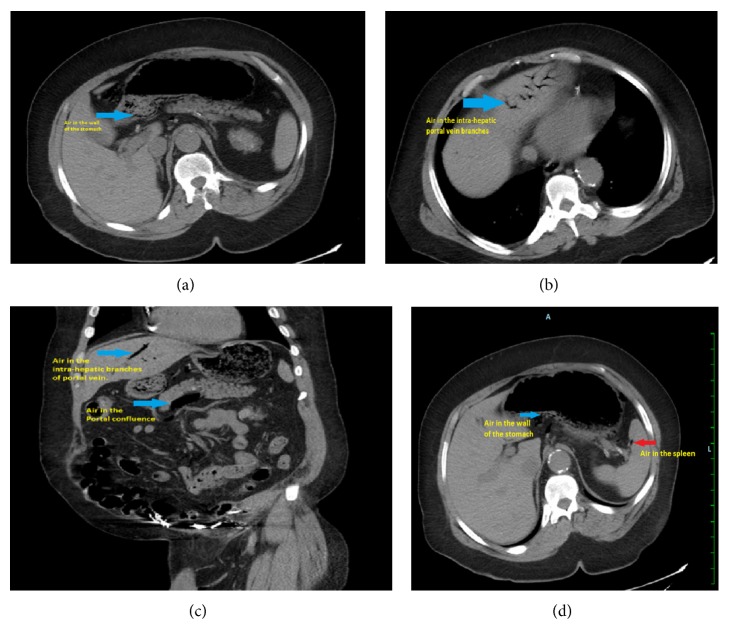
(a) Blue arrow showing air in the wall of the stomach. (b) Blue arrow showing air in the intrahepatic branches of portal vein. (c) Coronal section of the abdomen with upper arrow showing air in the intrahepatic branch of portal vein and lower arrow showing air in the area of confluence of portal vein. (d) Blue arrow showing air in the stomach wall and red arrow showing air in the spleen.

Patient had an elevated white blood cell (WBC) count to 13.4 k/mm^3^ (Normal range: 4–11 k/mm^3^). Lactic acid level was elevated to 2.7 mmol/L (normal range: 0.7–2.2 mmol/L). Serum amylase, serum lipase, and rest of the comprehensive metabolic panel were within normal limits. Subsequently, a computerized tomography (CT) scan of the abdomen was obtained which revealed air in the wall of the stomach along with large amount of air in the liver, spleen, portal vein, superior mesenteric vein, and splenic vein (Figure 1). However, there was no intestinal obstruction, intra-abdominal free air, or free fluid. Surgery was consulted as gastric pneumatosis and portal venous gas raised the suspicion of intestinal ischemia and bowel necrosis.

**Figure 2:**
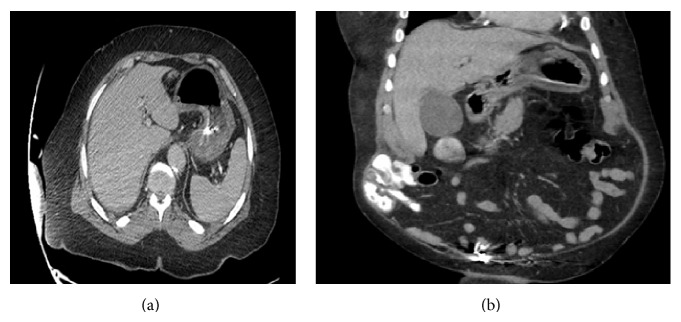
Contrast enhanced CT scan of the abdomen (48 hours after presentation) showing resolution of gas in the wall of the stomach, portal veins, and spleen.

In spite of the worrisome findings on the CT scan, patient was hemodynamically stable and abdominal examination was completely benign. Therefore, he was treated conservatively with bowel rest, gastric decompression, intravenous fluids, and antibiotics. He improved significantly over the next 2 days. A repeat CT scan of the abdomen (obtained after 48 hours from presentation) showed complete resolution of gastric pneumatosis and portal venous air (Figure 2). The proposed mechanism of these radiologic findings was that repeated episodes of vomiting and retching led to gastric emphysema in our patient, with subsequent dissection of air into the portal venous system. He was started on a clear liquid diet with gradual advancement. Antibiotics were discontinued and he was discharged home in stable condition.

## 3. Discussion

There are two types of gastric pneumatosis, emphysematous gastritis and gastric emphysema [1]. Emphysematous gastritis may occur by direct inoculation of gas-producing bacteria into the gastric mucosa or by hematogenous spread.* Clostridium perfringens, Escherichia coli, Pseudomonas aeruginosa, Streptococci, Staphylococci,* and* Enterobacter* species are the most frequent causative agents [9]. Immunosuppression, diabetes mellitus, ingestion of corrosive substances, alcoholism, and nonsteroidal anti-inflammatory drugs ingestion are common predisposing factors. Affected patients are very ill with severe abdominal pain, peritoneal signs, and elevated WBCs, often resulting in a fulminant clinical course, shock, and a high rate of mortality [9, 10].

Unlike emphysematous gastritis, GE is noninfectious in origin and occurs primarily due to entry of intraluminal air into the wall of the stomach. It may be divided into three etiological categories: traumatic, obstructive, and pulmonary [1].

Traumatic GE is caused by transmural diffusion of air after a mucosal injury, which can occur during esophagogastroduodenoscopy (EGD), severe vomiting, cardiopulmonary resuscitation (CPR), or acute gastric dilatation caused by eating disorders [2–4, 6, 11]. Obstructive GE has been reported in patients with gastric outlet obstruction due to a variety of conditions. The increase in the intragastric pressure accompanied by damaged gastric mucosa permits the entry of air into the wall of the stomach. Gastric carcinoma, gastric volvulus, duodenal obstruction, and hypertrophic pyloric stenosis in children have been reported as causes of obstructive GE [1, 5]. Pulmonary GE is caused by alveolar rupture and air leaks, which then track through the mediastinum and dissecting downwards to reach the stomach wall [1].

Clinical manifestations in GE are usually nonspecific. Patients may present with nausea, vomiting, epigastric discomfort, or abdominal pain. Importantly, patients are almost always hemodynamically stable and do not show signs of acute abdomen. In general, it has a benign clinical course and resolves spontaneously without any clinical sequel [1–4, 6].

HPVG was frequently seen with bowel necrosis, small bowel obstruction, peptic ulcer disease, and intra-abdominal abscess [7]. Hence presence of gas in the portal venous system was often considered to represent an intra-abdominal catastrophic event. However, with advances in imaging techniques and increasing availability of CT scans, many benign cases of HPVG have been found. Ulcerative colitis, barium enemas, colonoscopy, liver transplantation, GE, and cardiopulmonary resuscitation represent such benign causes of HPVG. Conservative treatment is successful in many of these cases without surgical intervention [7, 8, 12, 13].

Hussain et al. have classified patients into three broad groups to simplify the treatment approach. First, patients with HPVG and signs of acute abdomen are best treated with emergent surgery. Second, patients with HPVG who lack clinical signs and demonstrate stable hemodynamic parameters should receive conservative management. Finally, patients with HPVG and uncertain clinical features should undergo endoscopy and/or diagnostic laparoscopy to rule out underlying bowel ischemia [13].

We believe that, in our case, repeated episodes of vomiting and retching caused damage to the gastric mucosa and sudden increase in the intragastric pressure. This led to dissection and leaking of air into the intramural layer of the stomach wall and subsequent spread to the portal veins, superior mesenteric vein, and splenic vein (Figure 1). Because the patient presented with benign physical exam and laboratory findings, a conservative treatment approach was chosen. Clinical improvement and spontaneous resolution of air within two days as confirmed by a repeat CT scan of the abdomen (Figure 2) further substantiated our theory that the etiology of visceral air was benign and not due to ischemia of the bowel or stomach. Hence, HPVG does not always indicate serious intra-abdominal pathology. It can be associated with benign conditions as described above. Also HPVG is just a radiological sign and it should not be solely relied upon in deciding between surgical intervention and conservative treatment; rather entire clinical picture including underlying etiology, clinical examination, and laboratory parameters should be taken into consideration [13, 14].

## 4. Conclusion

The presence of air in the wall of stomach may be due to GE, which is often self-resolving, or due to emphysematous gastritis, which heralds worse prognosis. Superimposed occurrence of air in the portal venous system often causes a dilemma when considering surgical options. Historically, presence of gas in the portal venous system was considered as an acute abdominal emergency and warranted urgent surgical intervention but recently many benign causes of GE and HPVG have been described in the literature. These new cases were not associated with serious abdominal pathology and did not require surgical intervention. Therefore, HPVG by itself should no longer be an indication for emergent surgical intervention.
